# When the Ignored Gets Bound: Sequential Effects in the Flanker Task

**DOI:** 10.3389/fpsyg.2012.00552

**Published:** 2013-01-02

**Authors:** Eddy J. Davelaar

**Affiliations:** ^1^Birkbeck College, University of LondonLondon, UK

**Keywords:** flanker task, cognitive control, conflict monitoring, sequential dependencies, associative learning, episodic binding

## Abstract

Recent research on attentional control processes in the Eriksen flanker task has focused on the so-called congruency sequence effect a.k.a. the Gratton effect, which is the observation of a smaller flanker interference effect after incongruent than after congruent trials. There is growing support for the view that in this paradigm, the congruency sequence effect is due to repetition of the target or response across trials. Here, results from two experiments are presented that separate the contributions of target, flanker, and response repetition. The results suggest that neither response repetition alone nor conflict is necessary to produce the effect. Instead, the data reveal that only flanker repetition is sufficient to produce congruency sequence effects. In other words, information that is associated with a response irrespective whether it is relevant for the current trial is bound to response representations. An account is presented in which the fleeting event files are the activated part of the task set in which flankers, targets, and response representations are associatively linked and updated through conflict-modulated reinforcement learning.

## Introduction

Cognitive psychological research has shown that when a participant is instructed to make a response to a target stimulus, he or she is typically slower and less accurate when distractors are present. This is even the case despite receiving instructions to ignore these distractors and having had extensive practice on trials with targets and distractors. Theorists who address this distractor interference effect generally refer to these tasks as conflict or congruency tasks. However, there is great disagreement about how participants exert cognitive control in these tasks. Although some theories have been presented in great detail, by using computational methods, ambiguities still remain. One such computational theory of cognitive control in conflict tasks (Botvinick et al., 2001) has had such a substantial impact on the field that it spawned a plethora of investigations focusing on the precise neurocognitive mechanisms underlying conflict-modulated cognitive control and questioning the sufficiency and necessity of conflict to observe cognitive control.

Research has largely focused on three types of conflict tasks. In the Stroop (1935) task (for a review see MacLeod, 1991), participants are instructed to name the color of a word as quickly and accurately as possible. The word itself refers to a color, allowing the creation of congruent words, such as the word “green” in green font color, and incongruent words, such as the word “blue” in red font color. The second task is the Simon or spatial compatibility task (Simon and Rudell, 1967), in which a stimulus (letter, word, or symbol) is presented on the left or right side of the computer screen. The participant is required to press a left or right button based on the stimulus content while ignoring the stimulus location. For example, a congruent trial could be the word LEFT presented on the left side of the screen and an incongruent trial could be the word LEFT on the right side of the screen. Finally, in the flanker task (Eriksen and Eriksen, 1974), a central target character is flanked by distractors. There are more distractors than targets and the distractors may be identical to the target (congruent trial) or different than the target. In response-incongruent (RI) trials, the identity of the distractors is associated with the opposite response as the target.

These three tasks have largely been treated as identical in terms of the control processes involved, which has led to theorists making inferences and predictions about one task based on published findings in another task. In fact, the influence of stimulus repetitions differs greatly between the Stroop and flanker tasks (Mayr et al., 2003). In addition, the comparisons of response time distributions are fundamentally different (Spieler et al., 2000; Pratte et al., 2010), which may reflect differences in perceptual, response, and control processes. Thus, even though much of the research on conflict processing has used the Stroop and Simon tasks, those results can not readily be assumed to hold for flanker tasks. As it turns out, the flanker task is the odd one out when it comes to the effects of repetition of stimuli across trials (as will be discussed below) and is the only task that consistently falsified a necessary prediction of the conflict/control-loop model (Botvinick et al., 2001). This has led to a number of new models of conflict-modulated cognitive control that appeal to additional control processes (Blais et al., 2007; Verguts and Notebaert, 2008; Davelaar and Stevens, 2009). This paper continues this approach and addresses the relative influence of distractors on congruency sequence effects in the flanker task.

**Figure 1:**
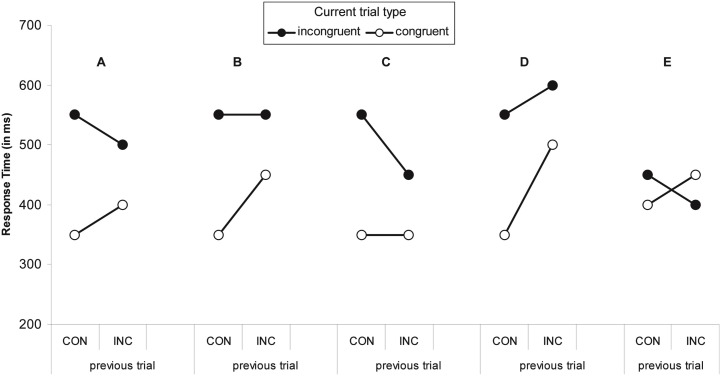
**Five interaction patterns with the same interaction effect [RT_cI−_RT_cC−_ (RT_iI−_RT_iC_)]**. The previous trial type is either congruent (CON) or incongruent (INC). The interaction effect is the observation of larger flanker interference effect (RT_incongruent−_RT_congruent_) after CON trials than after INC trials. The original explanations of congruency sequence effects explain pattern **(A)**. The same interaction effect size can be obtained with patterns that have no sequence effects for incongruent **(B)** or congruent **(C)** trials. Pattern **(D)** indicates worse control for incongruent trials after an incongruent trial. Patern **(E)** shows a congruency reversal. Pattern **(C)** can be obtained in Stroop tasks, whereas pattern **(E)** is found in Simon tasks.

Our starting point is the work by Botvinick et al. (1999, 2001); Kerns et al., 2004; see also Davelaar, 2008a). In several studies using the Stroop and flanker tasks, they observed that the anterior cingulate cortex (ACC), a frontal brain structure, is more activated in response to processing incongruent than congruent stimuli. Their theoretical innovation was that the ACC may be monitoring the amount of conflict in a trial and at the system level this measured conflict is used to enhance the amount of control on the next trial. Thus, the more conflict on trial n, the more control on trial *n* + 1. This would lead to a particular interaction called the congruency sequence effect or the Gratton effect, which has been interpreted as a signature of cognitive control in conflict tasks. The congruency sequence effect (Gratton et al., 1992) is the finding of a lower interference effect after an incongruent trial compared to the effect after a congruent trial (see Figure 1A). Congruent trials after an incongruent or congruent trial are referred to as iC and cC trials, respectively, whereas incongruent trials after an incongruent or congruent trial are referred to as iI and cI trials. The Botvinick-model explains congruency sequence effects as follows. On trial n, the incongruent stimulus leads to an increase in conflict, which is detected by the ACC. On trial *n* + 1, this increased conflict leads to more control, causing distracting information to be ignored more efficiently. Thus, incongruent (iI) and congruent (iC) trials will be responded to more quickly and more slowly, respectively. Although this pattern is observed in all three conflict tasks, several unresolved issues remain.

The first unresolved issue is that not all interactions between the previous and current trial type are created equal. Figures 1B,C show examples of an interaction pattern with the same interaction effect (100 ms). In Figure 1B, there is no control over incongruent trials, whereas in Figure 1C, the congruent trials are unaffected (cf., Kerns et al., 2004). Figure 1D has the same interaction effect, but would not fit the theoretical description of a Gratton effect. Finally, Figure 1E presents another interaction with the same effect size, but in this case there is a reversed interference effect after incongruent trials. This pattern is impossible to obtain with the Botvinick-model, as the theoretical limit is the complete absence (or perfectly ignoring) of distractors, which would lead to equal response times for iI and iC trials.

The reversal depicted in Figure 1E is most often found in the Simon task (Hommel et al., 2004) and is readily explained within the feature-integration account of sequential effects in the Simon task (Hommel et al., 2004). Specifically, the account assumes that stimulus and response representations on a trial get bound into a single representation called an event file. When part of the stimulus-response ensemble is repeated the remaining parts are reactivated. In the original account, subsequent trials that involve stimuli that partially match (and partially mismatch) the content of the event file lead to confusion and thus longer response times. Response times to stimuli that completely match or completely mismatch are assumed not to differ. Applied to the flanker task, the following is expected based on the feature-integration account. Assume that the stimuli are left- and right-pointing arrows, such as <<<<< and >>>>> as congruent trials and <<><< and >><>> as incongruent trials. To distinguish between <<<<< followed by <<<<< and <<<<< followed by >>>>>, the reference to the trials include whether the response repeats (e.g., cCr) or alternates (e.g., cCn; “*n*” for non-repetition). When the response repeats across trials, iIr and cCr trials are complete repetitions and will lead to faster response times compared to cIr and iCr trials. Dependent on the overall flanker interference effect, the final pattern will either resemble Figures 1A,E. The pattern is the same when the response alternates across trials. For example, iIn and cCn trials are complete mismatches whereas iCn and cIn are incomplete mismatches. Evidence in favor of the feature-integration account comes predominantly from studies employing the Simon task (Hommel et al., 2004) and supports the claim that specialized conflict-related processes (as assumed in the Botvinick-model) are not necessary to explain the congruency sequence effect.

Despite the success of the feature-integration account, both it and the conflict/control-loop hypothesis require further extensions in order to explain the pattern of sequential effects in the flanker task, which constitutes the second unresolved issue. Specifically, in the flanker task, the two-way interaction that resembles Figure 1A is found only when the response/target repeats across trials. When the response/target changes, the interaction is absent with the effects of the previous and current trial on response times being completely additive (i.e., parallel lines; Mayr et al., 2003; Nieuwenhuis et al., 2006; Bugg, 2008; Davelaar and Stevens, 2009)^1^. Interestingly, the original results reported in Gratton et al. (1992) reveals the same three-way interaction, but this was not analyzed. There is some variation across experiments with the effect of previous trial congruency with iCn and iIn being (equally) slower than cCn and cIn, respectively, which is due to an increase in the response threshold after a conflict trial (Davelaar, 2009).

To date, despite the introduction of new models of the flanker task (Hübner et al., 2010; White et al., 2011; see for debate, Hübner and Töbel, 2012) no computational theory has been put forward that accounts for this three-way interaction pattern. However, variations on the Botvinick-model with and without some elements of the feature-integration account have been proposed to address Stroop and Simon tasks (Blais et al., 2007; Verguts and Notebaert, 2008). These were then falsely assumed to also account for findings in the flanker task. An account that would be able to capture the three-way interaction pattern was given by Davelaar and Stevens (2009) and is essentially a weaker version of the feature-integration account. In particular, the assumption is that only complete matches lead to faster response times, whereas complete mismatches are equal to incomplete matches.

Even though the account put forward by Davelaar and Stevens (2009) captures the three-way interaction, it does not specify the relative importance of repeating the target, response, and flankers. In other words, in the feature-integration account relevant features are bound into an event file, but Davelaar and Stevens (2009) did not state what does and what does not get bound. At first blush, one would assume that the relevant features are the target and the response, but this would merely produce faster responses when the target/response repeats. Clearly, the flankers, despite being destined to be ignored, are included in the event files. The fact that they are not at all ignored is obvious from the existence of the flanker interference effect. What is not obvious is how the flankers are bound in the event file and what role they play in congruency sequence effects. To address this, we need a detailed account of how the task set or task instructions get represented by a participant. As will become clear in the next section, a very specific interpretation of the term “event file” is used together with a feature binding account of task representations.

### A feature binding account of task set representations

**Figure 2:**
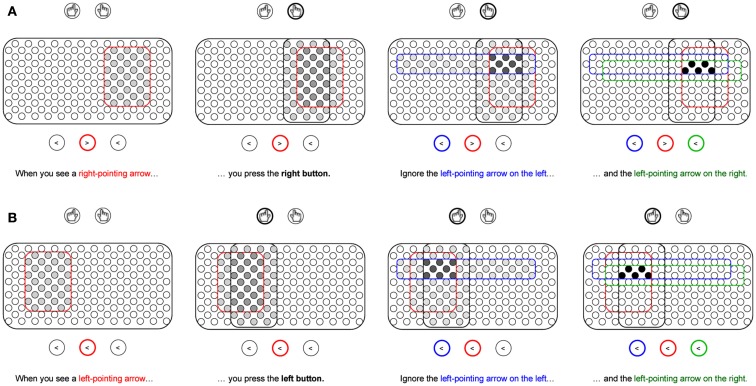
**Development of task representations in the flanker task**. **(A)** instruction for incongruent trials. **(B)** instruction for congruent trials. When the instructions are given, a subset of a pool of uncommitted neurons gets activated. With every new phrase in the instruction, overlapping pools of neurons are activated, but only those neurons in the overlapping region remain active and eventually become the representation of the instructed task rule. In the flanker task, target, response, and flanker information are bound into a single task representation. The example instructions are consistent across the two congruency conditions, even though in practice the flankers need not be ignored in congruent trials.

In typical laboratory settings, experimental paradigms present the participant with novel combinations of stimuli and responses. In order for the participant to follow the instructions required for the experimental task, an internal representation of the task is required in the form of task goals or task sets. These representations encode the task rules and can thus be assumed to correspond to a set of IF-THEN statements. Initially, these rules are maintained in declarative memory until the task becomes well-practiced and transferred to procedural memory. According to Davelaar (2011), goal representations are bindings of representations related to the stimuli and responses. The representation that binds the various subcomponents can be likened to an event file. However, the process by which the event file is formed may require two levels of associative learning. Figure 2 illustrates the creation of two rules that are employed in the flanker task, using the analogy of neurons in a brain area, presumably the prefrontal cortex (Dehaene et al., 1998). A pool of non-specific neurons exist that have the latent ability to form connections with other intra-pool neurons and with extra-pool representations, such as motor representations (indicated by circles with hands) and location-specific stimulus representations (indicated by circles with arrows). Let us assume that weak random connections exist from every extra-pool representation to the neuronal pool. When an instruction is given, extra-pool representations activate the units in the pool that happen (by chance) to be connected to them. In the example, the sentence phrase “When you see a right-pointing arrow…” activated the 31 units within the red enclosure. The next sentence phrase “…you press the right button” activated the units in the black enclosure. Importantly, only the units that were activated both during the first and second phrase will remain active and have pre-existing, albeit weak, connections with the extra-pool representations of the middle right-pointing arrow and the right motor program. The instructions also mention ignoring the left-pointing arrows on the left and the right. Instead of excluding these from the final representation of 5 units, they are integral to development of the goal representation. The 5 units will be strongly active, which lead to strengthening of the associative connections among them (see Figure 2; first level of associative learning) and with the extra-pool representations (second level of associative learning). The newly formed representation needs both levels of associative learning to be effective as a task representation. The intra-pool connectivity leads to a process of pattern completion: whenever one of the units becomes active, the entire assembly becomes active.

The initial fragile new representation will continue to strengthen during the practice trials and be sensitive to reinforcement signals. Computational theories of instruction learning and task goal representations typically use slow reinforcement-based learning algorithms (Dehaene et al., 1998; Sutton and Barto, 1998; Doll et al., 2009) and are thus only applicable at stages after initial creation of the task representation. This includes conflict-modulated learning (Davelaar, 2009). By using the pool of uncommitted neurons, a novel task representation can easily be created and kick-start the development of a more stable representation that eventually will lead to automatization of the task. As the stimuli differ in the amount of conflict they trigger, an asymmetry is expected whereby the task representations of incongruent stimuli compared to congruent stimuli undergo more changes in connectivity. The fate of distracting information is interesting, as it is associated with a response, but contextualized via the target. In the example, left-pointing distractors are included in both the top and bottom representations, but are associated with different responses (right and left, respectively). This perspective contrasts with views that ignored flankers would be associated with a “do-not-respond” tag as assumed in some theories of in negative priming (Neill et al., 1992; see for reviews, Mayr and Buchner, 2007; Schrobsdorff et al., 2012). The issue of putative negative priming in the flanker task will be discussed in the discussion.

In the account put forward here, the verbal instructions give rise to task representations, which through associative learning become strengthened further. This particular representation remains available during the entire experiment and increases in internal and external connectivity. Thus, this is a weight-based representation that stabilizes over protracted time. This contrasts with the fleeting event file referred to by Hommel and colleagues. According to their view, the event file resulting from feature-integration processes is of a transient nature (Colzato et al., 2006; Hommel and Colzato, 2009). These approaches are not incompatible. In fact, they form part of a continuum of various binding processes (cf. Colzato et al., 2006) that operate over different time-scales. In particular, one could consider the possibility that the transient event file could be the activated part of the task set and other salient activations. In other words, the transient event file may be the activated task representation. This distinction between a short-lasting activation-based representation and a longer-lasting weight-based representation has a long history in the memory literature (e.g., James, 1890; Norman, 1968; Shiffrin, 1976; Cowan, 1988) and computational theories (e.g., Davelaar et al., 2006) have explicated the interactions between these levels.

In the current study, the focus is on the presence of flanker information in the task representation and therefore flanker information will be present in the fleeting event file which influences responses in subsequent trials. This leads to two predictions. First, repeating flankers across trials should contribute to response repetition effects irrespective of whether the target repeats across trials. The flankers will continue to activate the previous task representation, which should facilitate complete reactivation. This is investigated in Experiment 1. Second, in the absence of responses on the previous trial, flankers should still prime the task representations, resulting in response facilitation on the following trial. This is investigated in Experiment 2 and is a consequence of the asymmetry in connection strength for incongruent and congruent task representations. Finding such a pattern of results supports the view that flankers are bound together with target and response information into a unique task representation and that this task representation is sensitive to cross-trial reactivations. This view is congruent with the findings that associative learning processes (as involved in creating the intra- and extra-pool connections) and the binding processes underlying event file creation (activation level of task representations) are different (Colzato et al., 2006).

### Sequential effects: What needs to be repeated?

The Eriksen flanker paradigm has provided critical insights into the spatiotemporal processes involved in visuospatial attention (Eriksen and Eriksen, 1974). Yet, the observation of cross-trial dependencies has attracted a lot of attention. This may be partly due to the explicit detail in prominent conflict/control theories (Botvinick et al., 2001) and partly due to leading contender theories (Hommel et al., 2004) that challenge core assumptions about the need to invoke conflict-related processes. The three-way interaction observed by Mayr et al. (2003) suggested that stimulus/response repetition was all that was needed to account for the pattern without recourse to conflict control processes. The early version of the repetition view was silent with regard to whether the target or the response needs to be repeated to obtain a congruency sequence effect. Take for example the arrow-flanker task used by Mayr et al. (2003). The transition <<><< to <<><< repeats the response, the target character and the flankers. Is it necessary to repeat all elements or is one or a combination of two (e.g., target and response) sufficient? To address this question one needs a flanker paradigm in which the target, flankers, and response can be manipulated independently. In the first experiment, a letter-flanker task is used, in which a consonant/vowel categorization is to be made on the central letter and has three types of stimuli: stimuli in which flankers (i.e., the distractors) are identical to the target (e.g., AAAAA: congruent; CO), different from the target but from the same category (e.g., EEAEE: stimulus-incongruent; SI), or are from a different category than the target (e.g., KKAKK: RI). The general finding is that RI-trials are slower than either CO- and SI-trials (Eriksen and Eriksen, 1974; Miller, 1991). The first experiment will make use of this task. The second experiment will use arrows instead of characters. In this arrow-flanker task, congruent, and incongruent trials are those in which the flanking arrows point in the same (e.g., <<<<<, >>>>>) or different (e.g., <<><<, >><>>) directions than the central target arrow. In the arrow-flanker task, the incongruent trials are both SI- and RI.

Using a letter-flanker task, response- and target-repetitions can be disentangled. It is possible that merely repeating the response is sufficient to speed up responding to a RI-stimulus after a RI-trial (e.g., KKAKK followed by BBEBB) compared to the same RI-stimulus after a CO-trial (e.g., AAAAA followed by BBEBB). Davelaar and Stevens (2009) reported an analysis from a study using a consonant/vowel categorization task, showing that the two-way interaction effect (previous x current trial type) was only observed when the target and flankers repeated across trials. Although this supports the view that the entire stimulus array needs to be repeated across trials to observe facilitation, it is also consistent with the view that flankers need to be repeated (Frings et al., 2007) together with the response. If a two-way interaction is found when flankers repeat while targets change (e.g., KKEKK followed by KKAKK), the repetition view needs to be updated to include the possibility that the flankers, despite being irrelevant (cf. Jaswal, 2012), are bound in episodic memory (i.e., activates the task representation) and can drive the sequential effects.

In a study that obtained the Gratton effect (i.e., the two-way interaction between previous and current trial type) when stimuli do not repeat across trials, Notebaert and Verguts (2006) proposed that stimulus conflict could contribute to the effect. Stimulus conflict is present when the flankers and target are different characters that are associated with the same response. Their study employed a numerical flanker task, which differs conceptually from the arrow-flanker task used in the original study by Mayr et al. (2003). The consonant/vowel categorization variant, as used here in Experiment 1, is conceptually closer due to the small set size (four letters versus two arrows versus ten digits) and a direct manipulation of stimulus conflict. It is possible that stimulus conflict, which is also present in RI-trials, underlies the conflict-modulated effect, as suggested by Verbruggen et al. (2006). This is yet unknown for a flanker paradigm using letters instead of numbers (Notebaert and Verguts, 2006), or colors (Verbruggen et al., 2006).

Finally, several researchers have suggested computational accounts in which monitored conflict modulates associative strengths (Blais et al., 2007; Verguts and Notebaert, 2008; Davelaar, 2009; Davelaar and Stevens, 2009). Evidence supporting this view comes from finding a larger speed up for repeated RI stimuli than for repeated CO stimuli (Davelaar and Stevens, 2009). Comparing RI- with CO-trials necessarily confounds stimulus-conflict with response conflict. The consonant/vowel variant of the flanker task deconfounds these factors and allows an assessment of the relation between type of conflict and the priming effect.

The present investigation aims to contribute to the literature by addressing the following questions. First, can the three-way interaction that supported the original repetition view by Mayr et al. (2003) be replicated in a consonant/vowel flanker paradigm? Second, what type of repetition (target, flanker, response, or a combination) is needed to obtain the two-way interaction between previous and current trial type? Answers to these questions provide critical boundary conditions for models of cognitive control and those that focus on the flanker task in particular. Specifically, knowing what type of information needs to be repeated will force the theorist to develop models that explicitly process this information. To preview the results, the observation that repeating the flankers and response are necessary suggests that flankers are not simply ignored, but form an integral part of any ensuing control process. Experiment 1 uses the letter-flanker task, while Experiment 2 uses an arrow-flanker task that singles out a critical pattern found in Experiment 1 and is predicted by the feature binding account of task representations.

## Experiment 1

### Materials and methods

#### Participants

Twelve participants (six women, mean age = 26) from the University of London were tested individually and received a remuneration of £7 for their time.

#### Stimuli and procedure

Stimuli consisted of five horizontally arranged capital letters. The letters used were: A, E, B, and K. The letters were arranged to create three types of stimuli: congruent (CO: AAAAA, EEEEE, KKKKK, BBBBB), stimulus-incongruent (SI: EEAEE, AAEAA, KKBKK, BBKBB), and RI (RI: BBABB, KKAKK, BBEBB, KKEKK, AABAA, EEBEE, AAKAA, EEKEE). To avoid biases in expecting a subset of stimulus transitions, all possible stimulus transitions were included. Stimuli were presented in black font on a white background. Participants were instructed to respond to the central target letter by pressing the “z” or “/”-key on the keyboard when the letter is a consonant or a vowel. The category-response mapping was counterbalanced across participants. The instruction was followed by a practice block of 48 trials (16 trials per condition). Each trial started with five dashes in gray font for 1,000 ms followed by the flanker stimulus presented for a maximum of 1,500 ms. Following the practice, participants completed 12 experimental blocks testing each condition 32 times. All blocks were followed by feedback regarding the accuracy and average response time. Participants were instructed to aim for an average response time of less than 1 s and to maintain accuracy above 80% correct.

### Results

Across participants and conditions, accuracy varied between 88 and 99%. There was a main effect of condition in both accuracy [*F*(2,22) = 14.33, MSE < 0.001, *p* < 0.001, η^2^ = 0.56] and correct RTs [*F*(2,22) = 66.63, MSE = 55.77, *p* < 0.001, η^2^ = 0.86]. Accuracy was lowest (92%) and correct RTs were slowest (518 ms) in the RI condition. Accuracy (95 vs. 96%) and correct RT (486 vs. 490 ms) did not differ between CO and SI conditions (all *p*s > 0.08). In addressing the two questions set out in the introduction, repeated measures ANOVAs were conducted on the accuracies and the RTs conditioned on the previous and current trial being correct (a standard procedure in this literature). As the various repetition effects do not allow a full factorial analysis, analyses were focused on the relevant parts of the data that address the questions. Table 1 presents correct RTs and error rates for all conditions (see Table 2 for examples). For correct RTs, an overall 3 (previous trial type) × 3 (current trial type) × 3 (repetition status) factorial ANOVA that included trials with flanker repetitions revealed main effects of current trial type [*F*(2,22) = 39.45, MSE = 650.89, *p* < 0.001, η^2^ = 0.78] and repetition [*F*(2,22) = 62.81, MSE = 2087.41, *p* < 0.001, η^2^ = 0.85], a two-way interaction between previous and current trial type [F(4,44) = 7.83, MSE = 357.31, *p* < 0.001, η^2^ = 0.42], and a three-way interaction [*F*(8,88) = 4.33, MSE = 425.49, *p* < 0.001, η^2^ = 0.28]. For error rates, there were main effects of previous trial type [*F*(2,22) = 6.05, MSE = 0.001, *p* < 0.01, η^2^ = 0.36], current trial type [*F*(2,22) = 14.66, MSE = 0.001, *p* < 0.001, η^2^ = 0.57], and repetition [*F*(2,22) = 20.35, MSE = 0.004, *p* < 0.001, η^2^ = 0.65], but no three-way interaction. The interactions in the RT data can be understood through addressing the questions, to which we turn now.

**Table 1:** **Mean correct response times (in ms) and error rates (in brackets), separated by flanker repetition**.

Transition	Previous trial type								
	No repetition			Response repetition			Target + response repetition		
	CO	SI	RI	CO	SI	RI	CO	SI	RI
RI	541 (0.06)	544 (0.09)	546 (0.11)^b^	**518** (0.11)	516 (0.10)	**528** (0.07)	**475** (0.03)^b^	469 (0.02)	**462** (0.04)^b^
RI-repeat^a^	524 (0.08)^bd^	527 (0.04)^d^	536 (0.07)^f^			**489** (0.05)^e^			**437** (0.03)^e^
SI	504 (0.05)^c^	505 (0.05)^c^	506 (0.05)		479 (0.05)	486 (0.03)	458 (0.03)^c^		452 (0.01)
SI-repeat^a^			511 (0.09)^d^	495 (0.04)				433 (0.02)^c^	
CO	503 (0.06)^bc^	501 (0.04)^c^	506 (0.06)	473 (0.05)	471 (0.04)	491 (0.06)		430 (0.01)^c^	437 (0.02)^b^
CO-repeat^a^			506 (0.11)^bd^				418 (0.01)^bc^		

*CO, congruent; SI, stimulus-incongruent; RI, response-incongruent*.

*^a^X-repeat refers to the X-condition in which the flankers repeat from the previous trial. ^b^These eight conditions are used in the analysis in question 1 on response conflict. ^c^These eight conditions are used in the analysis in question 1 on stimulus conflict. ^d^The effect of flanker repetition on RTs was not significant (*p* > 0.09 for all pairwise comparisons). ^e^The effect of flanker repetition on RTs was significant (*p* < 0.05 for all pairwise comparisons). ^f^This trial is a RI–RI transition in which the flankers do not become the target. The difference with the corresponding negative priming type transition was not significant (*p* > 0.55)*.

*The bold values feature in the target vs. flanker repetition analysis*.

**Table 2:** **Examples of stimuli used in Experiment 1 by condition**.

Transition	Previous trial type								
	No repetition			Response repetition			Target + response repetition		
	CO BBBBB	SI BBKBB	RI AABAA	CO EEEEE	SI AAEAA	RI BBEBB	CO AAAAA	SI EEAEE	RI BBABB
RI	KKAKK	KKAKK	BBABB^b^	BBABB	BBABB	KKAKK	BBABB^b^	BBABB	KKAKK^b^
RI-repeat^a^	BBABB^b^	BBABB	BBEBB^d^			BBABB			BBABB
SI	EEAEE^c^	EEAEE^c^	EEAEE		EEAEE	EEAEE	EEAEE^c^		EEAEE
SI-repeat^a^			AAEAA	EEAEE				EEAEE^c^	
CO	AAAAA^bc^	AAAAA^c^	EEEEE	AAAAA	AAAAA	AAAAA		AAAAA^c^	AAAAA^b^
CO-repeat^a^			AAAAA^b^				AAAAA^bc^		

*CO, congruent; SI, stimulus-incongruent; RI, response-incongruent*.

*^a^X-repeat refers to the X-condition in which the flankers repeat from the previous trial. ^b^These eight conditions are used in the analysis in question 1 on response conflict. ^c^These eight conditions are used in the analysis in question 1 on stimulus conflict. ^d^This trial is a RI–RI transition in which the flankers do not become the target*.

#### Question 1: is there a three-way interaction?

**Figure 3:**
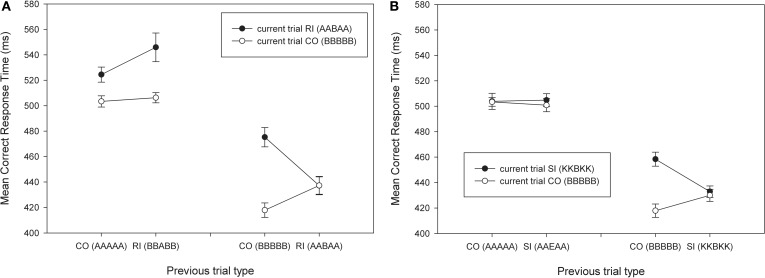
**Mean correct RTs as a function of current trial type and previous trial type**. **(A)** CO/RI combinations that include response conflict. **(B)** CO/SI combinations that include only stimulus conflict. Examples of previous and current trials are presented in brackets. CO, congruent; SI, stimulus- incongruent; RI, response-incongruent.

Figure 3A presents the correct RTs across the CO/RI conditions for which the transitions involve both response and target-repetitions. These conditions constitute the typical conditions used in previous experiments and involve response conflict and stimulus conflict. Replicating previous results, the two-way interaction is present for CO- and RI-trials, when the target and the response repeat, but is absent when there is no repetition. A repeated measures ANOVA crossing the factors current trial (CO vs. RI), previous trial (CO vs. RI), and repetition (no repetition vs. full repetition, including flankers) revealed a significant three-way interaction in the RT data [*F*(1,11) = 11.82, MSE = 731.31, *p* < 0.01, η^2^ = 0.52]. As expected this three-way interaction was due to a previous × current trial type interaction when target/response repeated [*F*(1,11) = 34.90, MSE = 283.01, *p* < 0.001, η^2^ = 0.76], which was absent when repetition was absent [*p* = 0.21, η^2^ = 0.14]. This replicates the three-way interaction previously reported in experiments using arrows as stimuli.

The remaining analyses take advantage of the task mapping two letters onto a single response, i.e., the SI conditions and flanker-non-repetitions.

Figure 3B presents the correct RTs across the CO/SI conditions for which the transitions involve both response and target-repetitions. These conditions do not involve any response conflict, but do have stimulus conflict. A similar repeated measures ANOVA using these CO and SI-trials revealed a three-way interaction [*F*(1,11) = 14.14, MSE = 179.05, *p* < 0.005, η^2^ = 0.56], which was due to a previous × current trial type interaction when target/response repeats [*F*(1,11) = 22.62, MSE = 187.74, *p* < 0.01, η^2^ = 0.67], but not when the target/response changes [*p* = 0.67, η^2^ = 0.02]. In other words, the data reveals a previous × current trial type interaction effect in the absence of response conflict.

#### Question 2: what type of repetition is needed to obtain the Gratton effect?

**Figure 4:**
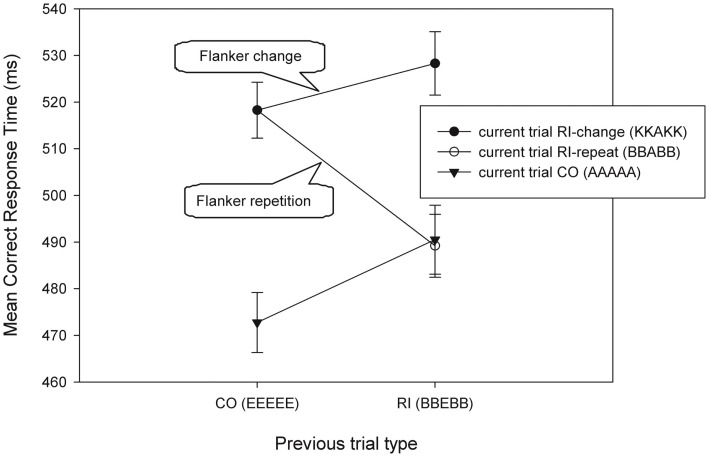
**Mean correct RTs as a function of current trial type and previous trial type for trial sequences that have response repetition, but no target repetition**. The RI-RI transitions can be split into trials that have flanker repetition (BBEBB followed by BBABB) and trials that have not (BBEBB followed by KKAKK). Examples of previous and current trials are presented in brackets. CO: congruent; RI: response-incongruent.

In both analyses focusing on response and stimulus conflict, both the target and response repeated across trials. In addition, the repeated CO–CO, SI–SI, and RI–RI transitions also repeated the flankers from one trial to the next. To address question 2, the focus is on Figure 4, which contains transitions that do not repeat the flankers. We conducted a 2 × 2 ANOVA on the data (with CO and RI as previous trial types) with the RI–RI transition that includes flanker repetition (e.g., BBEBB followed by BBABB) and one that includes flanker change (e.g., BBEBB followed by KKAKK). There was no previous × current trial type interaction effect when the flankers changed (RI–RI transition = 528 ms; *p* = 0.68, η^2^ = 0.02), but there was with flanker repetition (RI–RI transition = 489 ms) [*F*(1,11) = 9.4, MSE = 698.76, *p* < 0.05, η^2^ = 0.46].

No flanker repetition effects were found when the target and response change across trials (all *p*s > 0.09). With regard to the priming effects following different types of conflict, the RI-priming effect is numerically larger than the SI-priming effect (38 ms vs. 25 ms), but the sizes are not statistically different (*p* > 0.10).

### Discussion

The results can be summarized as follows. There are four effects in the data: an overall flanker interference effect (EEEEE vs. BBEBB), a response repetition effect (EEEEE followed by BBBBB vs. EEEEE followed by AAAAA), a combined target and response repetition effect (KKEKK followed by BBABB vs. KKEKK followed by BBEBB), and a flanker repetition effect (KKEKK followed by BBABB vs. KKEKK followed by KKAKK). Whereas none of these effects are controversial, the combination of these produces the three-way interaction effect between previous trial, current trial, and target/response repetition that has been discussed in the cognitive control literature (Mayr et al., 2003; Nieuwenhuis et al., 2006; Davelaar and Stevens, 2009). Importantly, the current experiment used a methodology involving letters, a non-spatial categorical judgment, and multiple target characters mapping onto a single response. Thus, the three-way interaction can not be attributed to the use of arrows as stimuli and confounding target repetition with response repetition.

We set out to address two questions. First, can the three-way interaction be replicated in a consonant-vowel flanker paradigm? Second, what type of repetition (target, flanker, response, or a combination) is needed to obtain the Gratton effect? The answer to the first question is unequivocally “Yes!.” In addition, the results show for the first time that this three-way interaction is present in both CO/RI combinations and CO/SI combinations. Therefore, the interaction is not dependent on response conflict. However, this description in terms of response and stimulus conflict is qualified by the findings to the second question. The analyses revealed that when nothing repeats across trials, the two-way interaction between current and previous trial type, which defines the Gratton effect, is absent. The interaction is also absent when only the response repeats. To obtain the two-way interaction at least the flankers and the response need to be repeated across trials. This finding supports the view that flanker repetition (in the absence of target repetition) contributes to the Gratton effect. Previously, the flanker benefit has been demonstrated by Frings et al. (2007) using a negative priming paradigm. Here, flankers are shown to critically contribute to congruency sequence effects that were previously attributed to global attentional control processes.

In relation to the conflict monitoring theory (Botvinick et al., 2001), the results revealed that conflict by itself does not produce a Gratton effect and that stimulus repetition seems to govern its presence. The experiment did not show a Gratton effect after RI and SI conflict when nothing repeats across trials. In one study, Verbruggen et al. (2006) found a marginal interaction effect between previous trial type (CO vs. SI) and current trial type (CO vs. SI), but did not report any statistics or effect sizes. In following Davelaar and Stevens (2009), the size of the RI-priming effect (RT_EEEEE followed by KKEKK−_RT_KKEKK followed by KKEKK_) was larger than the SI-priming effect (RT_EEEEE followed by AAEAA−_RT_AAEAA followed by AAEAA_). Within the associative learning theory that uses conflict-modulated learning (Davelaar and Stevens, 2009; see also Blais et al., 2007; Verguts and Notebaert, 2008) this could be interpreted to mean that response conflict is a stronger learning signal than stimulus conflict. However, the statistics did not support the numerical difference. This aspect would benefit from further research.

## Experiment 2

In the first experiment, flanker repetition contributed to the previous × current trial type interaction when the response also repeated. This is consistent with a theoretical view in which traces are formed in episodic memory during the instruction phase and contain information about the flankers and the response. Target information would also be stored in the trace. These episodic traces are different, but related, to what Hommel (1998; Hommel et al., 2004) refers to as event files. At the current stage in the theoretical development, it is assumed that these episodic traces form the task representations and that short-lived event files are the activations of these representations. In other words, the relation between event files and task representation is analogous to the relation between short-term memory and long-term memory within the activation-based approach (e.g., James, 1890; Norman, 1968; Shiffrin, 1976; Cowan, 1988). This reinterpretation of event files allows for integration with the literature on memory and executive function (Davelaar et al., 2005; Davelaar, 2011) and underscores the breadth of binding processes proposed (Colzato et al., 2006). Whether the activation-based approach can be distinguished from Hommel’s event files depends on the definitions and characteristics attributed to them. Nevertheless, in the current incarnation as activated task representations, any bound element, including flankers, could lead to congruency sequence effects.

To truly assess whether flanker repetition contributes to the congruency sequence effect, only flanker information should be presented. In Experiment 2 some of the trials are preceded only by flankers. As there is no target, no response is needed. If the task representation includes flanker information, then merely presenting flankers should lead to (partial) reactivation of those representations and subsequently to (pseudo)repetition effects. This prediction follows from the observation that task rules are activated despite being unnecessary for a given trial (see Hommel et al., 2004, for a review). Congruency sequence effects have been observed when no response on the previous trial was made (Hommel et al., 2004, experiment 3), indicating that features presenting in a preceding trial can activate task rules that have their influence on subsequent trials.

Critical in the current theorizing is the assumption that the strengthening of the intra- and extra-pool connections is modulated by reinforcement and conflict signals. Thus the more conflict, the larger the change in connection strength (Davelaar, 2009). This leads to an asymmetry, whereby the task representations for incongruent stimuli have stronger connections than those for congruent stimuli. This means that flankers tend to activate the incongruent task representation more than the congruent one.

Consider the four possible scenarios: >> >> followed by >>>>>, >> >> followed by >><>>, >> >> followed by <<<<<, and >> >> followed by <<><<. The flankers >> >> do not predict the stimulus on the following trial, but this set of flankers has stronger connections with the task representation “IF >><>> THEN left button” than with “IF >>>>> THEN right button.” This produces a competitive advantage that leads to faster responses when the stimulus >><>> is presented. Thus, even though no target or response has occurred on the previous trial, the content of the event file includes the activated task representation, which in turn facilitates responses on the next trial when a stimulus matches that representation. This is tested in Experiment 2.

### Materials and methods

#### Participants

Eighteen participants (12 women, mean age = 29 years) for the University of London were tested in individually and received a remuneration of £8 for their time.

#### Stimuli and procedure

Stimuli consisted of five horizontally arranged arrowheads, making up congruent (<<<<< and >>>>>) and incongruent (<<><< and >><>>) trials. Stimuli were presented in black font on a white background. Participants were instructed to respond to the central target arrowhead by pressing the “z” or “/”-key on the keyboard when the arrow pointed to the left or the right, respectively. On 25% of the trials the target would be absent (blank space) and participants should withhold responding for 1,000 ms. This produced four new sequences: incongruent and congruent trials that were preceded by similar or different flankers. The instruction was followed by a practice block of 64 pairs of trials. Each pair of trials started with a blank interval for 1,000 ms followed by the first flanker stimulus, presented for a maximum of 1,500 ms, followed by another blank interval and finally the second flanker stimulus. From the viewpoint of the participant, each block consisted of 128 independent trials. Feedback on accuracy and reaction time was given after the practice block and after each experimental block. Participants were instructed to aim for an average response time of less than 1 s and to maintain accuracy above 80%. After the practice block, participants completed eight experimental blocks, testing each unique responding condition 32 times and each of the sequences with flankers only trials 64 times.

### Results

**Figure 5:**
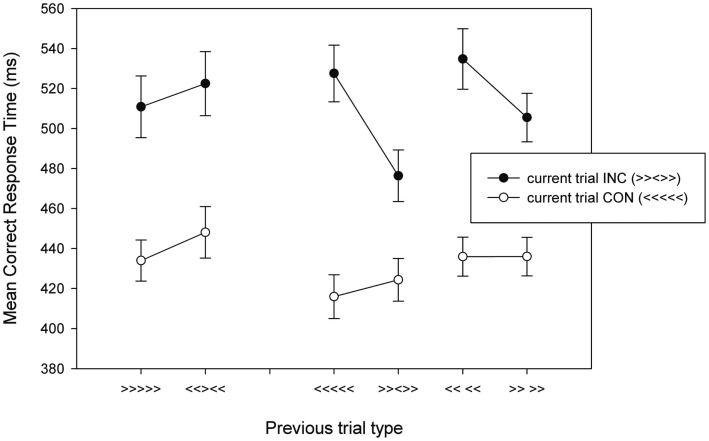
**Mean correct RTs as a function of current trial type and previous trial type for Experiment 2**. Examples of previous and current trials are presented in brackets. INC, incongruent; CON, congruent.

A 2 × 2 × 2 within-subject ANOVA with previous trial type (repeating response versus non-repeating response), current trial type, and target/response repetition as factors was conducted, followed by a 2 × 2 ANOVA on the no response flanker repetition trials. The results are presented in Table 3 and Figure 5. Incongruent trials were slower and less accurate than congruent trials [RT: *F*(1,17) = 142.41, MSE = 1565.81, *p* < 0.001, η^2^ = 0.150; error: *F*(1,17) = 22.39, MSE = 0.005, *p* < 0.001, η^2^ = 0.568]. There was a main effect of previous trial type on error rates [*F*(1,17) = 13.36, MSE = 0.002, *p* < 0.005, η^2^ = 0.440], but not on RTs (*p* > 0.10) and a main effect of repetition for RTs [*F*(1,17) = 17.41, MSE = 656.11, *p* = 0.001, η^2^ = 0.506], but not for error rates (*p* = 0.612). All two-way interactions for error rates were significant [previous × current: *F*(1,17) = 110.03, MSE = 0.001, *p* < 0.01, η^2^ = 0.371; previous × repetition: *F*(1,17) = 6.05, MSE = 0.003, *p* < 0.05, η^2^ = 0.263; current × repetition: *F*(1,17) = 10.22, MSE = 0.001, *p* = 0.005, η^2^ = 0.375], and all but the current trial type × repetition interaction for RTs [previous × current: *F*(1,17) = 26.13, MSE = 331.35, *p* < 0.001, η^2^ = 0.606; previous × repetition: *F*(1,17) = 25.42, MSE = 414.32, *p* < 0.001, η^2^ = 0.599; current × repetition: *p* = 0.287]. These interactions were qualified by significant three-way interaction in both the RTs [*F*(1,17) = 20.41, MSE = 359.31, *p* < 0.001, η^2^ = 0.546] and error rates [*F*(1,17) = 11.85, MSE = 0.002, *p* < 0.005, η^2^ = 0.411]. These three-way interactions are due to the presence of a previous × current trial type interaction when the target/response repeats [RT: *F*(1,17) = 40.42, MSE = 394.90, *p* < 0.001, η^2^ = 0.704; error rate: *F*(1,17) = 14.41, MSE = 0.003, *p* = 0.001, η^2^ = 0.459], but not when it changes (*p*s > 0.37). These results replicate the basic pattern presented in previous reports (Mayr et al., 2003; Nieuwenhuis et al., 2006; Davelaar and Stevens, 2009).

**Table 3:** **Mean correct response times (in ms) and error rates (in brackets), separated by previous trial type, current trial type, repetition, and flanker only trials**.

Current trial type	Previous trial type					
	No repetition		Target/response repetition		Flankers only	
	Congruent	Incongruent	Congruent	Incongruent	Change	Repeat
Incongruent	511 (0.05)	522 (0.06)	528 (0.12)	476 (0.03)	535 (0.07)	506 (0.04)
Congruent	434 (0.02)	448 (0.01)	416 (0)	424 (0)	436 (0.01)	436 (0.01)

In this experiment, the trials that were preceded by flankers only and did not require any response are the main focus. A 2 × 2 ANOVA revealed a significant effect of current trial type [*F*(1,17) = 134.14, MSE = 950.30, *p* < 0.001, η^2^ = 0.888] and an effect of flanker repetition [*F*(1,17) = 26.69, MSE = 133.69, *p* < 0.001, η^2^ = 0.628]. Importantly, these main effects were qualified by a significant interaction [*F*(1,17) = 27.38, MSE = 141.18, *p* < 0.001, η^2^ = 0.617], which was due to an effect of flanker repetition on incongruent trials [*t*(17) = 5.93, *p* < 0.001], but not on congruent trials (*p* > 0.98).

The final statistical comparison that tests the view that the three-way interaction is due to flanker repetition is to correlate the two-way interaction in repetition trials (*M* = 58.9, SD = 9.6) with the flanker repetition effect in the incongruent trials (*M* = 25.1, SD = 2.7). This correlation was significant (*r* = 0.63, *p* < 0.01) and suggested that 40% of the variance in the two-way interaction is accounted for by flanker repetition in the incongruent trials. In order to put this finding in perspective, all four pairwise effects (flanker effect in flanker change and flanker repetition trials and flanker repetition effect in congruent and incongruent trials) were entered as predictors for the critical two-way interaction effect. The overall multiple regression was significant [*F*(3,13) = 4.68, MSE = 988.84, *p* < 0.05, *R*^2^ = 0.52]. Of the predictors, only the flanker repetition effect in incongruent trials remained significant [*t*(13) = 2.60, *p* < 0.05] and accounted uniquely for 34.1% of the variance in the two-way interaction effect. The other predictors were not significant (*p*s > 0.23). As a final check, when a two-way ANOVA was run with factors previous trial type and current trial type for the repetition only trials (iCr, iIr, cCr, cIr) with the flanker repetition effect in incongruent trials entered as a covariate, the two-way interaction was not significant [*F*(1,15) = 0.015, MSE = 269.10, *p* = 0.905, η^2^ = 0.001].

### Discussion

Experiment 2 provides strong evidence for the view that repetitions of flankers partially drive the previous × current trial type interaction. First, it shows that there is no need to make a response or even to see a target in order to get an interaction. Importantly, the non-necessity of a response and a target on the preceding trial shows that response conflict and stimulus conflict are not necessary to observe a speed up in response times in incongruent trials. Second, it places the locus of the effect squarely on processing of incongruent trials. Theories that attribute the congruency sequence effect only to conflict-related processing will have difficulties accommodating these findings unless other mechanisms are included.

One such mechanism, as suggested by one reviewer, could be negative priming, which is known to influence response times on subsequent trials in the absence of responding on a preceding trial or trial frame. Presenting the flankers on the first trial will help the participant learn about their irrelevance and thereby tagging them as such in a “conventional” event file that binds the flankers to an internal “ignore” response. Of course this scenario implies that feature bindings in an event file can include representations that are neither stimulus-related nor response-related, but are “cognition”-related. That is, event files may include bindings to cognitive states. Extending the breadth of what is bound in an event file is one direction of current research efforts (Colzato et al., 2006; Hommel and Colzato, 2009), but even this version fails to capture the entire data set. In particular, if an internal “ignore” response is bound to the flankers, the presentation of a stimulus with target and flankers constitutes a partial match. According to the feature-integration account, the sequences with flanker only trials with flanker repetitions should be slower than those with flanker change, which constitute complete mismatches. If we make a different arbitrary assumption that “ignore” responses do not contribute to mismatch calculations, but lead to actually ignoring the flankers in the subsequent trial, incongruent trials should become faster and congruent trial become slower. Although this is an interaction that matches Figure 1A, it is not the interaction found in the experiment, which matches Figure 1C.

It is not inconceivable that a specific feature-integration account can be given for this particular finding, but the scenario put forward here is the following. The assumption is that flankers are part of task representations that include response information. Therefore when >> >> is presented both the “IF >>>>> THEN right button” and the “IF >><>> THEN left button” task representations are activated with a competitive advantage for the latter. Only when flankers repeat is the corresponding response (i.e., pressing the left button) in the most active task representation facilitated. This results in an interaction between flanker repetition and congruency that is entirely driven by flanker repetition in incongruent trials. In addition, this facilitation underlies the congruency sequence effect in the sequences with complete stimuli, as shown by the regression analyses.

## General Discussion

The current experiments addressed the question whether flankers, despite the requirement to be ignored, are influencing cross-trial congruency effects, as manifest by an interaction between previous and current trial type: the congruency sequence a.k.a. the Gratton effect. In Experiment 1, the Gratton effect was observed when only flankers and responses were repeated across trials. This finding demonstrated the necessity of flanker repetition, together with the non-necessity of target repetition and response conflict. Experiment 2 further revealed that flanker repetition alone is sufficient to produce congruency sequence-like effects, suggesting that no conflict (stimulus or response) or an actual response is needed to produce the effect. Moreover, using regression analyses, it was shown that the congruency sequence effect is fully accounted for by this flanker repetition effect. This is not to say that the conflict-related mechanisms suggested in the literature do not play a role. Statistically, there is another 65.9% of the variance to be accounted for, but with 34.1% only due to flanker repetition this can not be ignored. However, given that the congruency sequence effect disappeared when controlling for the interaction due to flanker repetition, the current hypothesis is that conflict does not contribute directly to the effect.

The experiments put important constraints on current theorizing. Egner (2007) reviewed the literature on conflict tasks and addressed the two leading alternative explanations: conflict adaptation and feature-integration. Both of these theories are able to account for the two-way interaction, but for different reasons. In the former, the interaction is due to conflict-triggered adjustment of attentional focus. In the latter, the interaction is due to speed up of complete matches and mismatches. As mentioned in the introduction, the data shows that the target/response repetition is a modulating factor. Neither theory accounts for this modulating effect, but a minimal extension to the feature-integration account suffices. For example, facilitation could be assumed to occur only for complete matches and not for complete mismatches. Although this would capture the data, it would not explain why in the flanker task complete mismatches do not lead to facilitation, whereas in the Simon task they do.

The theoretical view put forward here assumes that task representations are formed at the beginning of the experiment and are activated during the experiment when stimuli are presented. The activation of the task representation persists after a trial is terminated, setting up a bias, or expectation for the required response in the next trial. Response times in the next trial are facilitated when the stimulus matches the active task representation. The strength of the associative connections within the task representation and with the stimulus and response representations increase with continued use according to the associative learning rules, which are sensitive to reward and conflict. According to this view merely repeating the flankers is sufficient to activate the task representations and thereby set up expectations about the required response on the next trial. The experiments presented here question the role of conflict of any type in congruency sequence effects. Instead, the proposal is that conflict has an indirect influence by being used as a learning signal in ongoing stabilization and proceduralization of task representations. Other pathways through which conflict indirectly influence cognitive control are outlined elsewhere (Davelaar, 2009).

A recurrent critique to associative learning accounts of the Gratton effect is that the RI–RI transition in the no repetition situation (e.g., BBABB followed by AABAA) promotes negative priming (Ullsperger et al., 2005; Bugg, 2008). Bugg (2008) compared the critical trials against a neutral baseline and found slower RTs for the RI–RI transition, which was interpreted to provide support for a negative priming effect counteracting the expected conflict-induced speed up. However, two types of evidence argue against this interpretation. First, in a similar experiment, no such increase in RT was found (Davelaar and Stevens, 2009). Second and more importantly, “a similar magnitude of slowing was also observed” (Bugg, 2008; p. 1221) on RI–RI and RI-CO transitions (in the letter notation that would be: BBABB followed by BBBBB) relative to the neutral conditions. Clearly, if negative priming is evident for the RI–RI transition, the same influence of the previous flankers should produce positive priming for the RI-CO transition. Such a pattern was never observed in the literature that focuses on sequential effects in the Eriksen flanker task (see figures in: Mayr et al., 2003; Ullsperger et al., 2005; Nieuwenhuis et al., 2006; Verbruggen et al., 2006; Bugg, 2008; Davelaar, 2009; Davelaar and Stevens, 2009). This is not to say that negative priming can never be found with a flanker paradigm. Quite the opposite. Stadler and Hogan (1996) obtained negative and positive priming effects in a numerical flanker task. The main discrepancy between their findings and the aforementioned literature is that Stadler and Hogan (1996) exclusively employed stimuli in which the target and the flankers were associated with different responses. In other words, they only used RI-trials, which may trigger a stronger requirement and reliance on attentionally deselecting the flankers, producing negative priming effects. To date, there is no report showing evidence of negative priming in a binary flanker task that includes incongruent and congruent trials. Even in Experiment 1, which contained four types of RI–RI transitions, there was no evidence for slower RTs for transitions where the flankers became the target compared to transitions where flankers changed across trials (all *p*s > 0.5).

Another recurrent comment is that as a whole, the Stroop and Simon tasks do not show the three-way interaction and therefore the original conflict model need not require modification. This comment falsely implies that all three congruency tasks are equal. Several recent reports have documented differences in response time distributions. For example, Spieler et al. (2000) showed that the RT distributions of incongruent Stroop, but not flanker trials have a longer tail than the corresponding congruent trials. Pratte et al. (2010) contrasted the Stroop and Simon tasks, revealing that the Stroop effect increases with increasing quantiles of the RT distribution, whereas the Simon effect decreases with increasing quantiles. Davelaar (2008b) showed that RT distributions in the flanker task are sensitive to stimulus repetition, such that the flanker interference effect increases with increasing quantiles of the RT distribution, unless an incongruent stimulus repeats. Together these studies question the extent with which findings from one task can be assumed to be obtained in another.

In conclusion, the experiments presented here adds to the body of literature by demonstrating the considerable impact of flanker repetition in a phenomenon previously attributed to general attentional control processes. Despite the requirement of being ignored, the flankers reactivate task representations and thereby prime certain responses on the subsequent trial. Thus, flankers are bound in the representations that drive congruency sequence effects. In the flanker paradigm, monitored conflict might only have a modulatory role in adjusting associative connections of task representations. Future research, using the flanker task, could explore the boundary conditions of the repetition effects with regard to the nature of event files, the influence of task representations, and the process of conflict-modulated (indirect) control.

## Conflict of Interest Statement

The author declares that the research was conducted in the absence of any commercial or financial relationships that could be construed as a potential conflict of interest.

## Footnotes

^1^Some studies still reported an interaction when the target/response does not repeat across trials (Ullsperger et al., 2005; Notebaert and Verguts, 2006; Verbruggen et al., 2006). However, these studies used different methodologies (e.g., very short presentation durations or using a large set of numbers instead of two arrows) than the studies listed. Egner et al. (2010) observed an inverse correlation between the congruency sequence effect and the temporal separation between consecutive trials. This highlights the impact of seemingly arbitrary methodological choices, such as timing and stimulus material. The current work aims to address explicitly what underlies the three-way interaction between previous trial type, current trial type, and target/response repetition by using methods that are known to give rise to the three-way interaction. Future work could investigate why some studies failed to replicate this three-way interaction effect.
